# Ant social network structure is highly conserved across species

**DOI:** 10.1098/rspb.2024.0898

**Published:** 2024-07-31

**Authors:** Tomas Kay, Alba Motes-Rodrigo, Arthur Royston, Thomas O. Richardson, Nathalie Stroeymeyt, Laurent Keller

**Affiliations:** ^1^ Laboratory of Social Evolution and Behavior, The Rockefeller University, New York, NY, USA; ^2^ Department of Ecology and Evolution, University of Lausanne, Lausanne, Switzerland; ^3^ School of Biological Sciences, University of Bristol, Bristol, UK

**Keywords:** ants, social insects, collective behaviour, social network analysis, social organization, division of labour

## Abstract

The ecological success of social insects makes their colony organization fascinating to scientists studying collective systems. In recent years, the combination of automated behavioural tracking and social network analysis has deepened our understanding of many aspects of colony organization. However, because studies have typically worked with single species, we know little about interspecific variation in network structure. Here, we conduct a comparative network analysis across five ant species from five subfamilies, separated by more than 100 Myr of evolution. We find that social network structure is highly conserved across subfamilies. All species studied form modular networks, with two social communities, a similar distribution of individuals between the two communities, and equivalent mapping of task performance onto the communities. Against this backdrop of organizational similarity, queens of the different species occupied qualitatively distinct network positions. The deep conservation of the two community structure implies that the most fundamental behavioural division of labour in social insects is between workers that stay in the nest to rear brood, and those that leave the nest to forage. This division has parallels across the animal kingdom in systems of biparental care and probably represents the most readily evolvable form of behavioural division of labour.

## 1. Introduction

Social insects are among the most ecologically successful organisms on the planet. There is an estimated 12 Mt of dry carbon ant biomass on Earth—more than that of all wild mammals and birds combined [1]. Their ecological success is thought to result from their sophisticated social organization, which increases per capita productivity [2–4] and confers a range of other benefits including mitigating predation risk and increasing territory defence capability [5,6]. The study of social insect colony organization has recently been revolutionized by the advent of high-throughput behavioural data [7]. The extraction of the coordinates of hundreds of colony members multiple times per second for extended periods of time has allowed the automatic inference of social interactions and task-related behaviours [7–12]. These data have greatly advanced our understanding of colony social organization. For example, we now know that increased group size and genetic heterogeneity can confer fitness benefits by enhancing division of labour [2,13] (though do not always seem to [14]), how fundamental aspects of insect biology map onto colony social networks [15,16] and how social network structure is influenced by both pathogens [17,18] and symbionts [19]. We also know more about the dynamics of individual-level behavioural change [9,20,21], foraging and food dissemination [22,23], cooperative transport [24] and other aspects of self-organization [12,25–27], all of which are important topics for scientists investigating biological and artificial collective systems, and optimization algorithms [28–30].

**Figure 1.**
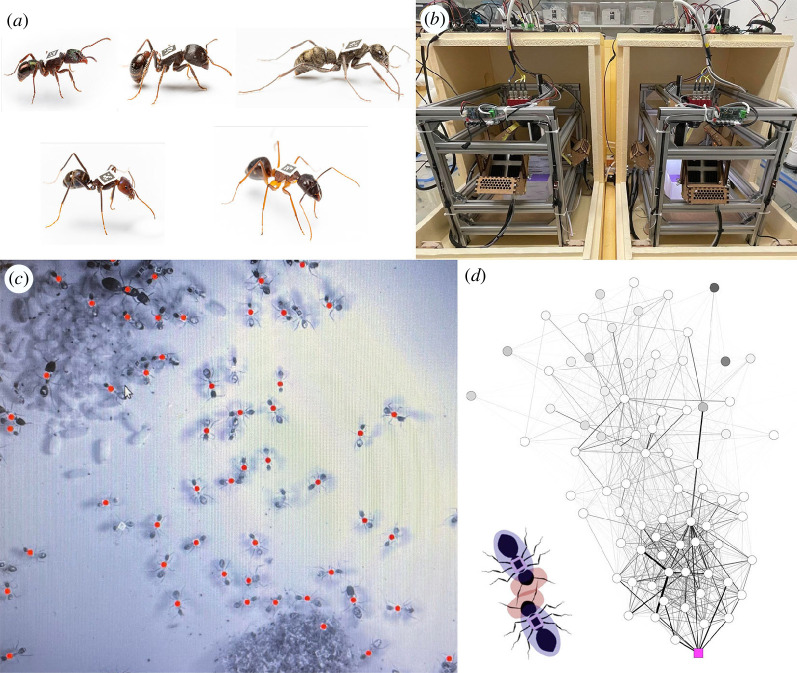
Experimental overview. (*a*) Single workers of each of the study species. Clockwise from top-left: *R. metallica*, *P rugosus*, *D. rugosum*, *C. fellah* and *I. purpureus*. Images not to scale. Photo credit: Bart Zijlstra. (*b*) The double-box tracking set-up comprises two boxes positioned within two tracking systems that can be independently climatically controlled to simulate a nest box and a foraging box. (*c*) The automated detection of tags inside the tracking system. Red dots are superimposed onto the tags that have been recognized. (*d*) An example *C. fellah* social network. Edge colour intensity and width correspond to pairwise interaction frequency (darker and thicker, higher frequency). Circular nodes represent workers (coloured according to the proportion of time they spent in the foraging arena: darker, more time foraging), while the magenta square represents the queen. Edges are weighted and node positions are defined by a force-directed layout meaning that ants that interact more frequently are positioned closer together. Inset is an illustration of how interactions were detected through the intersection of ‘head capsules’.

Studies investigating insect social organization have generally—though not exclusively [12]—used a single species to assess how one or few biological variables change in response to a given factor. Consequently, while social network structure (and its implication for colony function) is understood for certain species in detail, we know little about its natural variation among species. Ant social systems are tremendously diverse: there is variation in the number and type of reproductive individuals [31], the number and types of worker castes [32], mating system [33], nest structure [34], foraging strategy [35], system of food re-distribution [36], etc. It is possible that certain aspects of social network structure vary as a consequence of some of these traits, that certain aspects of network structure are directly selected according to the life history of the species and that other aspects of network structure are fundamental and invariant across taxa. Here, we take a first step towards addressing this knowledge gap by characterizing and comparing the social networks of five ant species (*Camponotus fellah*, *Iridomyrmex purpureus*, *Diacamma rugosum*, *Pogonomyrmex rugosus* and *Rhytidoponera metallica*; figure 1*a*) from five different subfamilies (Formicinae, Dolichoderinae, Ponerinae, Myrmicinae and Ectatomminae, respectively). While this sampling is too sparse to demonstrate how social network structure varies with any particular life-history trait, it is sufficient to establish which aspects of network structure vary across species, and which may be conserved. Moreover, we selected these particular study species because they naturally differ in various important life-history traits including the presence and number of queens, number of matings, mature worker population size, colony structure, colony founding and foraging behaviour. Observed differences provide a first indication as to the co-variation of life-history features and social network structure.

*Camponotus fellah* has a relatively standard life history for an ant: singly mated queens found colonies independently following mating flights [37,38]. Queens and workers are dimorphic and mature colonies naturally comprise approx. 10 000 workers [37]. *Pogonomyrmex rugosus* is broadly similar but differs importantly in that queens mate multiply, increasing genetic diversity among workers [39]. In *I. purpureus*, queens mate with one or few males, there can be many queens per colony, and colonies can spread across multiple nest sites and reach worker population sizes of up to 300 000 [40,41]. In both *R. metallica* and *D. rugosum*, workers can mate and become functional reproductives known as gamergates. In *D. rugosum,* the queen caste has been completely lost. Instead, each colony includes a single gamergate which physically sterilizes her daughter workers by removing thoracic appendages from them as they emerge as adults [42,43]. In *R. metallica*, the queen caste persists but is not always present within functional colonies [44,45]. Refer to table 1 for an overview of the biology of these species.

**Table 1.** Summary of the life history and ecology of the study species. (DD, data deficient.)

trait	*Diacamma rugosum*	*Camponotus fellah*	*Rhytidoponera metallica*	*Pogonomyrmex rugosus*	*Iridomyrmex purpureus*
reproductives	1 gamergate [42,43]	1 queen [37]	0—many queens + gamergates [44,45]	1 queen [37,46]	1—many queens [47,48]
matings	1 [42,43]	139	1—few [44]	3–1240	1—few [48,49]
workers	100 [50]	10 K [37]	30–1 K [51]	600–15 K [52,53]	11–300 K
colony structure	monodomous [43]	DD	DD	monodomous [37]	polydomous [40,41]
founding	probably budding [43]	independent [54]	independent + budding [44]	independent [37,55]	independent [48]
foraging	solitary [50]	DD	solitary	solitary + group [56]	DD

Here, we focus on two fundamental social network properties. The first is modularity, which measures whether and how the network segregates into clusters of individuals—known as modules or communities—that interact frequently among themselves and rarely with others. The second is (weighted) node strength, the total number of interactions that a given individual has during the experiment. These properties are critical to understanding the dynamic functioning of any network [57,58], and have been previously shown to relate to division of labour and colony performance [7,17,20]. We examine how these network features relate to reproductive and behavioural division of labour and explore some of the behavioural mechanisms governing these relationships. We find that the network structures are broadly similar across species. All species formed social networks that (i) exhibit community structure; and (ii) are best characterized as comprising two social communities—a ‘nurse community’ and a ‘forager community’. The extent of community structure correlates with the extent to which the species exhibits division of labour. We find that workers tend to be strongly affiliated to one or the other social community, and that the foraging community is larger than the nurse community in all species. Across species, the relationship between foraging behaviour and social network position is similarly nonlinear, and individuals that forage more tend to interact less. The latter finding seemingly stems from foragers roaming over larger and less densely populated areas within the nest than nurses. Finally, the queen’s position in the social network appears to be highly variable across species. Queens range from being no different to the average member of the nurse community to being stand-out ‘hub nodes’ at the centre of the nurse community to being peripheral to the nurse community and highly specialized in their interaction profile (i.e. interacting frequently with a small subset of nurses). This variation appears not to be driven by differences in queen space-use patterns but rather queen–worker encounter kinetics in terms of (i) the number of workers to encounter the queen per unit of time; (ii) the frequency with which those same workers encounter the queen; and (iii) the rate of turn-over in the identity of the workers that encounter the queen.

## 2. Results and discussion

We tracked five colonies of each species in double-box set-ups (i.e. with a constantly dark nest box and a foraging box subject to day–night condition cycles; figure 1*b,c*). Experimental subcolonies comprised the queen (all colonies in the laboratory were maintained with one queen), 100 randomly sub-sampled workers and a proportion of the total brood that approximately matched the proportion of total workers that had been sampled (i.e. if 100 workers was 50% of the work force then 50% of the brood was sampled). Data from across the 5 day period was pooled for static network construction and analysis. This design constrains the generality of our findings, but allows for effective comparison of the different behavioural tendencies of the five species in a common garden environment. Additionally, since the laboratory environment differs from the natural environment of each species in different ways, some of the observed species-level differences may reflect different responses to being in the laboratory.

### (a) Ant social networks comprise two communities

**Figure 2.**
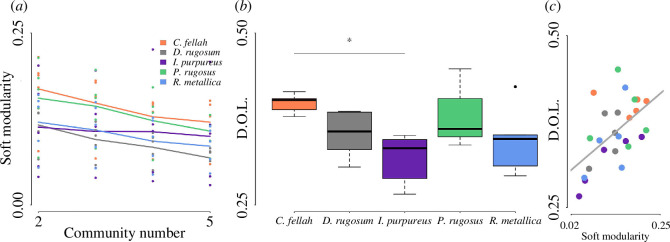
Community structure and division of labour. (*a*) On average, soft modularity (a measure of how well a proposed number of communities fits a given network) decreased with community number in all five species. Points represent individual colony values and lines represent species averages. (*b*) The extent of division of labour (D.O.L.), measured per colony as the standard deviation in proportion of time spent outside of the nest. An asterisk (*) indicates *p* < 0.05 in two-sample *t*-tests. (*c*) Across all colonies, network modularity correlates with division of labor (Pearson’s *r* = 0.553; *p* = 0.004).

**Figure 3.**
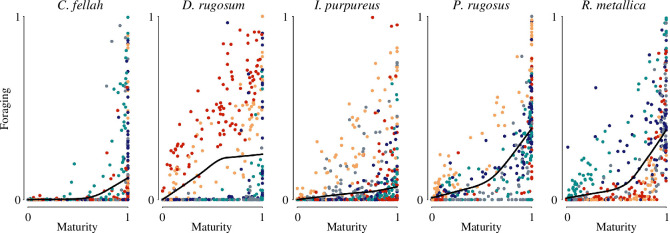
Community membership and foraging behaviour. In all species, there was a clear and nonlinear relationship between social maturity and foraging behaviour, and there appears to be a strong colony effect on the maturity at which workers start foraging. For example, foraging seems to only start increasing with maturity after a maturity of 0.8 in all five *C. fellah* colonies, while in two *D. rugosum* colonies foraging increases with maturity even at the lowest end of the maturity range. Colour indicates colony and lines represent locally estimated scatterplot smoothing regressions.

**Figure 4.**
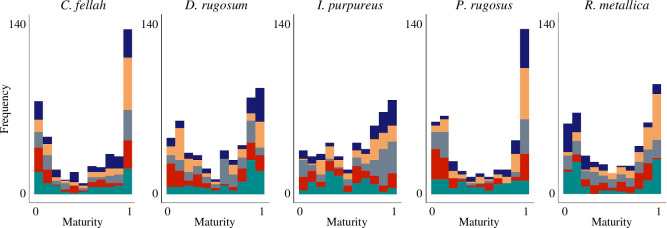
Distribution of workers across the two communities. In all species workers tended to be deeply embedded in one or the other social community. Colour indicates colony.

**Figure 5.**
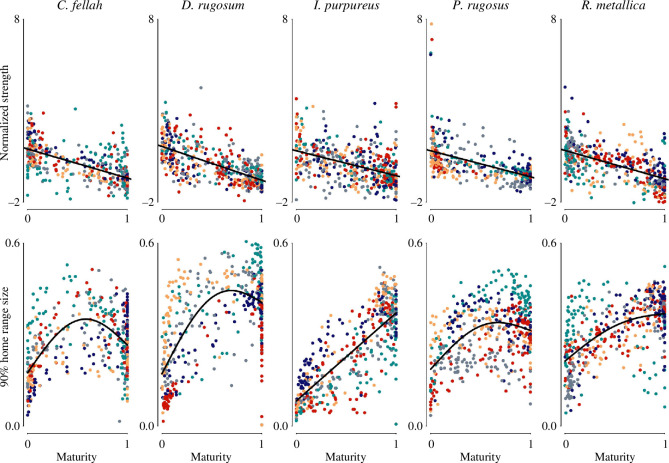
Node strength, social maturity and spatial dispersion. Top row: in all species, there was a quantitatively similar negative relationship between social maturity and node strength (i.e. interaction frequency) when node strength was normalized within colonies to have a mean of 0 and a standard deviation of 1. Bottom row: in all species, there was a positive correlation between social maturity and proportion of the nest box occupied by the 90% home range. Colour indicates colony and lines represent linear regressions (top row) and generalized additive models (bottom row).

To evaluate how a proposed number of social communities fits a given network we calculated soft modularity scores, maximizing and quantifying the ratio of within : between group connections [12,59]. The social networks (e.g. figure 1d–5; electronic supplementary material, figures S1–S5) of all species were, on average, significantly more modular than expected by chance (permutation test comparing each network against randomly rewired versions of the same network: all *p*-values < 0.001; electronic supplementary material, figure S6). The same was independently true for 22 out of 25 individual colonies, the exceptions being one *C. fellah* colony and two *D. rugosum* colonies. Furthermore, in all species, the networks were most parsimoniously partitioned into two communities: the cross-colony average soft modularity score peaked for all five species when community number was set to 2, and decreased monotonically as community number was increased to 5 (see figure 2*a*; electronic supplementary material, figure S7 for colony-level plots). The differences were statistically significant in paired *t*-tests for 2 versus 3 (*t* = 3.42; *p* = 0.027), 4 (*t* = 3.88; *p* = 0.018) and 5 (*t* = 5; *p* = 0.008) communities for *C. fellah*. The same was true for *D. rugosum*, while in *P. rugosus* and *R. metallica* the differences were significant only between 2 versus 4 and 5 communities, and none of the differences were significant for *I. purpureus*. While the presence of the two communities seems qualitatively consistent, there was considerable quantitative variation in how clearly segregated these communities were, with the most modular network (*C. fellah* colony 3 = 0.21) being five-fold more modular than the least modular (*I. purpureus* colony 3 = 0.04).

We next wondered whether colonies that exhibited more modular social networks also exhibited more pronounced division of labour. Since we measured only a single task (foraging), we used the colony-level standard deviation in the proportion of time spent foraging to quantify the division of labour at the colony level. A colony in which all individuals had foraged at exactly the same rate would have a score of 0, and the score would increase as foraging became more biased towards a subset of individuals. Variation in network modularity was associated with the extent of division of labour (figure 2*b*): colonies with more modular networks exhibit more pronounced division of labour (Pearson’s *r* = 0.553; *p* = 0.004; figure 2*c*). The species-level differences in the extent of division of labour were only statistically significant between *C. fellah* and *I. purpureus*, which exhibited the most and least division of labour respectively (Welch two-sample *t*‐test: *t* = 4.58, *p* = 0.005).

Previous work with *C. fellah* established that the two social communities correspond primarily to a division between nurses and foragers [7,20]. To test whether this was true for all species in this study, we quantified individual network positions using ‘social maturity’—a measure of community membership that ranges continuously from 0 to 1, with 0 representing an individual deeply embedded in the nurse social community, 0.5 representing an individual equally affiliated to both communities and 1 representing an individual deeply embedded in the forager community [20]. Since individuals shift from nursing to foraging as they age (‘temporal polyethism’), and move through the colony social network accordingly, their social maturity scores should increase over their lives. While we do not have data on nursing behaviour, the two communities consistently seem to separate foragers from nurses as all species exhibited a clear positive correlation between social maturity and the proportion of time spent in the foraging arena (species-level generalized linear mixed models (GLMMs) testing the relationship between foraging and social maturity, with colony as a random effect for *C. fellah*: *t* = 10.4, *p* < 0.001; *D. rugosum: t* = 16.5, *p* < 0.001; *I. purpureus: t* = 12.7, *p* < 0.001; *P. rugosus: t* = 22.0, *p* < 0.001 and *R. metallica: t* = 23.2, *p* < 0.001; figure 3). Across species, individuals that spent a considerable time in the foraging arena were almost always restricted to one of the two communities (i.e. the foraging community). The presence of two social communities in all five species implies that the segregation of the social network into nurse and forager communities is probably a fundamental characteristic of ant social networks.

### (b) Temporal polyethism and the distribution of individuals across communities

The distribution of workers between the two social communities reflects the dynamics of temporal polyethism. Long-term tracking of *C. fellah* showed that workers spend 80% of their lives strongly affiliated with either the nurse or forager community and 20% of their lives in transition between the two [20]. This was reflected in the fact that 20% of workers had intermediate maturity values at a given snapshot in time, as would be expected under stable demography.

We found that for all five species, there were more individuals with extremal than intermediate maturity values (one-sample proportions test with continuity correction for the numbers of individuals with maturity scores between 0.25 and 0.75 versus the numbers of individuals with maturity scores outside of that range: *p* < 0.01 for all species; figure 4).

This implies that, based on analogy with previously collected *C. fellah* data [20], workers of all species tend to spend a greater proportion of their lives strongly associated with one or the other community, and a smaller proportion of their lives in transition. While there were always less individuals with intermediate than with extremal maturity scores, there was considerable variation in the percentage of individuals with intermediate social maturities. The values were 20% for *C. fellah*, 27% for *D. rugosum*, 37% for *I. purpureus*, 19% for *P. rugosus* and 23% for *R. metallica*, with species identity having a statistically significant effect (one-way ANOVA, *F* = 2.97; *p* = 0.045). Higher proportions of intermediate individuals should reflect slower transitions from nurse to forager relative to worker lifespan. There was additionally a similar imbalance of workers between the two communities across species. In *C. fellah,* there were 44% more individuals with maturity scores of greater than 0.9 than with maturity scores of less than 0.1. The equivalent figure was: 53% for *D. rugosum*, 107% for *I. purpureus*, 65% for *P. rugosus* and 83% for *R. metallica*, although the variation within-species was large such that the variation between species was not statistically significant (one-way ANOVA, *F* = 0.394; *p* = 0.811). The balance of the workers between these two communities should reflect the average age at which workers transition, with younger transitioning species having comparatively more high-maturity workers.

### (c) Nurses interact more than foragers

It has previously been established that in *C. fellah*, nurses interact more frequently with other workers than do foragers (i.e. that strength decreases as social maturity increases), and we replicated this finding here (GLMM with strength as response variable, social maturity as explanatory variable and colony as random effect: *t* = −17.2; *p* < 0.0015). The same pattern held for the other four species (*D. rugosum*: *t* = −20.2, *p* < 0.001; *I. purpureus*: *t* = −11.1, *p* < 0.001; *P. rugosus*: *t* = −15.2, *p* < 0.001; *R. metallica*: *t* = −16.6, *p* < 0.001; figure 5). If this pattern resulted from foragers spending more time outside of the nest (where the density of individuals is lower), we would expect a positive association between total foraging output and the strength of association between community membership and node strength. However, while the average percentage of time that foragers (those that forage at least once) spent outside of the nest ranged from 1% in one *D. rugosum* colony up to 60% in one *C. fellah* colony, there was no relationship between these averages and the strength of correlation between social maturity and node strength (Pearson’s *r* = 0.011, *p* = 0.593; electronic supplementary material, figure S8*a*,*b*). This implies that foragers spending time in the foraging arena is not what makes their strength lower than nurses. The difference in strength between foragers and nurses instead seems to result from different space-use patterns within the nest, where foragers roam over larger areas, with lower ant density and encounter other ants less frequently (GLMM with social maturity as response variable, within-nest home range size as explanatory variable and species identity as random effect: *β* = 1.70; *t* = 30.2; *p* < 0.01). Interestingly, social interaction frequency seems to decrease with age in various other animals including red deer [60] and humans [61].

**Figure 6.**
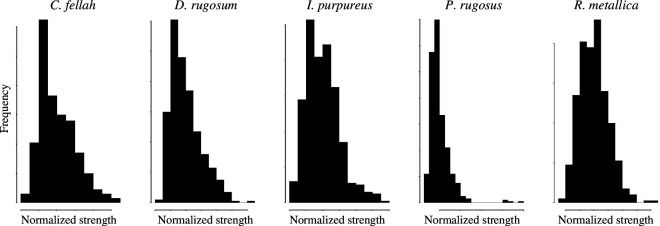
Degree distribution. The distributions of node strengths were consistent across species and were approximately log-normal.

More generally, the overall distribution of node strength is an important network property in determining the efficiency of information or resource spread and robustness to perturbation. Early work on biological networks suggested that they were generally ‘scale-free’ [62]. In scale-free networks, the distribution of node strength follows a power law, with highly connected ‘hub’ nodes, while in random networks node strength is normally distributed [63]. Hub nodes both increase the speed of transmission through the network and make it more failure-prone. The emerging consensus is now that scale-free networks are rather rare in nature and that node strength is typically normally distributed [64]. We find that the distribution of node strength is highly consistent across species, and follows an approximately log-normal distribution (i.e. somewhat intermediate between a normal and a power law distribution; figure 6).

### (d) Queens occupy unique social network positions

The dynamics of queen–worker interactions are subject to a number of potentially antagonistic evolutionary pressures. For example, there is evidence from various species that the queen, as the most valuable member of the colony, is kept socially isolated from certain other colony members to reduce the likelihood of her becoming infected with a pathogen [13,17]. Contrastingly, in various species, queens deliberately interact with many individuals to maintain or communicate their status—either through dominance interactions or the transmission of queen pheromone [65,66].

**Figure 7.**
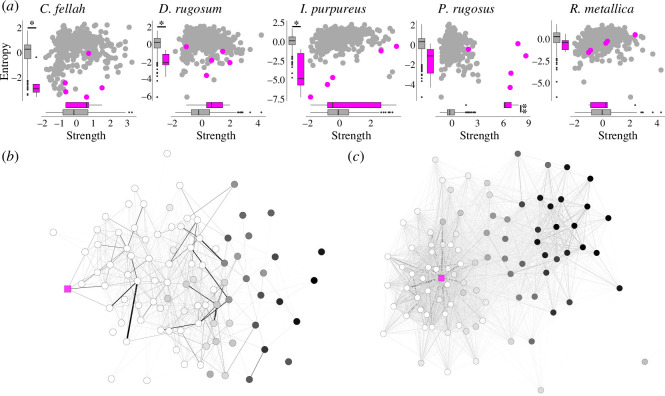
Queen role in the social network differs across species (*a*) Node entropy plotted as a function of node strength. Grey circles, workers; magenta circles, queens. Boxplots of queen and worker strengths and entropies are inset, and asterisks correspond to statistical significance in Welch two-sample *t*-tests. (*b*) An example *C. fellah* network (refer also to figure 1*e*), in which the queen has low entropy but average strength, and (*c*) an example *P. rugosus* network in which the queen has high strength but average entropy. Edge colour intensity and width correspond to pairwise interaction frequency (darker, more frequent). Circular nodes represent workers (coloured according to the proportion of time they spent in the foraging arena: darker, more time), while the square magenta node represents the queen.

We quantified the queen’s position in the social network with two parameters: node strength and entropy (i.e. how evenly distributed their interactions were across colony members, with low values indicating that queens interact frequently with a small subset of workers and high values indicating that queens interact at a similar frequency with all workers). The difference in social position between queens and workers varied considerably among species (figure 7). In *C. fellah*, *I. purpureus* and *D. rugosum*, queens/gamergates had lower entropies than workers but worker-typical strengths (i.e. they interacted as frequently as workers but were far more specific in their interaction partners; Welch two-sample *t*-tests comparing *C. fellah* queen versus worker normalized entropies: *t* = 4.49, *p* = 0.01; strengths: *t* = −0.675, *p* = 0.536; comparing *I. purpureus* queen versus worker normalized entropies: *t* = 3.36, *p* = 0.028; strengths: *t* = −0.608, *p* = 0.576, and comparing *D. rugosum* gamergate versus worker normalized entropies: *t* = 3.73, *p* = 0.02; strengths: *t* = −1.24, *p* = 0.28). Contrastingly, *P. rugosus* queens had entropies that were not significantly different from workers but had higher strengths (i.e. their interaction profile was not more specific than those of workers, but they interacted more frequently overall; Welch two-sample *t*-tests comparing *P. rugosus* queen versus worker normalized entropies: *t* = 2.12, *p* = 0.1; strengths: *t* = −5.41, *p* < 0.01). Finally, *R. metallica* queens were not statistically different from workers in either strength or entropy (Welch two-sample *t*-tests comparing queen versus worker normalized entropies for *R. metallica*: *t* = 1.51, *p* = 0.204; strengths: *t* = −0.365, *p* = 0.733). The similarity between the queen and worker network positions in *R. metallica* is consistent with this species’ reduced reproductive division of labour. *Rhytidoponera metallica* colonies typically contain multiple gamergates, with the queens being non-essential, sometimes absent, and possibly in the process of being evolutionarily lost [45].

The observed variation in queen strength and entropy could result from variation in queen space-use: queens with higher entropy (those that interact more evenly) may move around the nest box more to encounter more and different workers. To test this, we first discretized the arenas into tessellated hexagons of two ant body lengths, and then calculated the proportion of the nest box occupied by the queen’s 90% home range. Calibrating hexagon size with ant body length meant that the total number of hexagons per arena varied by colony/species, however this variation did not correlate with our home range size estimates (Pearson’s *r* = −0.193; *p* = 0.354). Despite considerable variation in queen home range size (12 ± 11% mean *±* s.d.), differences in queen movement do not correlate with queen entropy or strength (GLMM with entropy as the response variable, home range size as the explanatory variable and species identity as a random effect: *t* = 1.25; *p* = 0.227; GLMM with social strength as the response variable, home range size as the explanatory variable and species identity as a random effect: *t* = −0.754; *p* = 0.46; electronic supplementary material, figure S9*a*,*b*).

The observed variation in queen strength and entropy may alternatively result from the kinetics of queen-worker encounters independently of queen movement. For example, queen strength and entropy would be increased by more queen–worker interactions occurring at a given instant (either because worker attraction to queens is higher or because queens position themselves in areas of higher worker density). Entropy could be increased without increasing strength if the number of queen–worker interactions at a given instant did not vary, but there was a higher turn-over in the identity of workers involved in queen–worker interactions. Also finally, strength could be increased and entropy decreased by the same subset of workers engaging more frequently in interactions with the queen. We quantified (i) the average number of workers a queen contacts per hour; (ii) the average number of times she interacts with each of these workers; and (iii) the conservation in the identity of the workers with which she interacts from one hour to the next. These analyses revealed that queen strength was mostly determined by the frequency of contact between the queen and the workers. Contrastingly, queen entropy was determined by the number of workers contacted per hour and the conservation in the identity of the contacted workers from one hour to the next. Specifically, there was a significant association between the average proportion of the workforce that the queen contacts per hour and queen entropy (GLMM with species identity as a random effect: *t* = 2.48; *p* = 0.022; electronic supplementary material, figure S9*d*) and a statistically insignificant trend for queens with higher overall strength to interact with a larger proportion of the workforce in a given hour (GLMM with species identity as a random effect: *t* = 1.85; *p* = 0.077; electronic supplementary material, figure S9*c*). There was a strong positive correlation between the average number of interactions per worker per hour (excluding 0 values) and queen strength (GLMM with species identity as a random effect: *t* = 3.32, *p* = 0.003; electronic supplementary material, figure S9*g*) but the relationship with entropy was not statistically significant (*t* = −0.233, *p* = 0.818; electronic supplementary material, figure S9*h*). Finally, there was a significant association between conservation in identity of the workers with which a queen interacts from one hour to the next and queen entropy (GLMM with species identity as a random effect: *t* = −2.70; *p* = 0.013; electronic supplementary material, figure S9*f*). Together these results indicate that variation in queen network position is probably driven by a combination of the number of different workers that come into contact with the queen per hour, how frequently these workers contact the queen and how much turn-over there is in which workers come into contact with the queen. It would be interesting to study how the social network positions occupied by queens vary with the queen number in the species that naturally have multiple queens—variation that we miss here by constraining queen number to one for all colonies.

## 3. Conclusion

Our comparative analysis revealed striking qualitative similarity in social organization across five ant species, from five different subfamilies, which last shared a common ancestor over 100 Mya [67–69]. All species formed modular networks that were most parsimoniously divided into two communities—one comprising workers that stay within the nest to raise the young, and the other comprising workers that sometimes leave the nest to collect food. At any given moment, most workers were deeply embedded in one or the other community and few workers were similarly associated with both communities. The absence of qualitative differences in this social organization between species suggests that it has been maintained by selection. Moreover, the division between carers and foragers has independently arisen many times across the animal kingdom in systems of biparental and cooperative care from mongooses to humans [70,71], and probably represents the most readily evolvable form of division of labour.

Our results also highlight striking interspecific variation in the social network position of the queen. Queens seem to occupy one of the three social positions: (i) stand-out hub nodes at the centre of the nurse social community; (ii) peripheral to the nurse social community and highly specialized in their interaction profile; and (iii) no different to the average worker. These differences could relate to various aspects of queen biology: *C. fellah* and *P. rugosus* are monogynous species with large queen–worker dimorphism and their queens are most different from workers in social network position. In *D. rugosum*, where there is no queen–worker dimorphism and *R. metallica*, where there is low queen–worker dimorphism and queens are relatively unimportant, queens/gamergates occupy the most worker-like social network positions. It would be particularly interesting to see whether the observed difference between the social positions of *C. fellah* and *P. rugosus* queens are typical between the queens of Formicine and Myrmicine species, and how variation in queen–worker polymorphism and queen number relates to the social network positions of queens within genera where these traits are most plastic. More generally, broader taxonomic sampling would also allow us to test (i) whether the apparently conserved features identified here are conserved when more species (with more diverse life histories) are considered; and (ii) which environmental/biological variables drive the observed species-level variation in network structure.

## 4. Material and methods

### (a) Ant colonies

*Camponotus fellah* colonies were established from queens collected after mating flights in Tel Aviv, Israel in 2007 and 2011. *Pogonomyrmex rugosus* colonies were established from queens collected after mating flights in Arizona, USA in 2008 and 2013. *Iridomyrmex purpureus* and *R. metallica* colonies were established from queens collected after mating flights in Victoria, Australia in 2021. *Diacamma rugosum* colonies were field collected in Chiang Mai, Thailand in 2018. The colonies of the different species were reared in the laboratory under different temperature and humidity regimes according to their ecological preferences: *C. fellah* = 29°C, 60% humidity; *P. rugosus* = 30°C, 60% humidity; *D. rugosum*, *I. purpureus* and *R. metallica* = 26°C, 60% humidity. All colonies were subject to 12 L : 12 D cycles, provided water ad libitum and fed weekly with flies and honey solution (at approx. 5% concentration). *Camponotus fellah* and *P. rugosus* were additionally fed ad libitum with artificial ant food and a seed mix, respectively [72]. All colonies contained either a single functional queen or gamergate and had been kept in the laboratory for at least six months.

To control for colony size, 100 workers were randomly selected for tracking from each colony along with the queen/gamergate and approximately the same proportion of brood as the 100 workers constituted of the total worker population (our laboratory colonies of all species generally contained 200–500 workers).

### (b) Automated behavioural tracking

The colonies were tracked in double-chamber set-ups, with a nest box (169 mm × 223 mm for *C. fellah*, *D. rugosum*, *I. purpureus* and *P. rugosus* and 131 mm × 179 mm for *R. metallica*) kept in constant darkness and connected to a foraging arena (subject to 12 L : 12 D cycles) of the same dimensions through a plastic tube (internal diameter 19 mm for *C. fellah*, *D. rugosum*, *I. purpureus* and *P. rugosus* and 10 mm for *R. metallica*). A single test tube filled with water and bunged with cotton was placed into each box to provide a constant water source. Unique matrix barcodes (1.4 mm^2^ for *C. fellah*, *D. rugosum*, *I. purpureus* and *P. rugosus* and 1.0 mm^2^ for *R. metallica*) from the ARTag library [73] were glued to the thorax of each ant using SAUER skin adhesive (12% resin). This glue is designed to attach urinary sheaths to skin and the combination of natural latex and resin makes it non-toxic, water-resistant and keeps tags attached for longer than superglue, as it does not set brittle.

Colonies were continuously tracked for five full days (from 00.01 to 23.59) at 5 frames s^−1^. Three of the replicates (one *I. purpureus* and two *R. metallica*) terminated early owing to technical failures, but the reduction in data did not result in these colonies having outlier values for the measured metrics. The tracking system saves video files and the position and orientation of each tag in each frame. Full technical specifications and source code for the tracking systems [19,74] are available at: https://github.com/formicidae-tracker.

The tracking data were post-processed in FortStudio, where the head–tail axis (front edge of clypeus to tip of abdomen) of each ant was annotated to establish the deviation of the inherent tag orientation from the head–tail axis, and to estimate body length (electronic supplementary material, figure S10*a*). Head and body capsules were annotated around each tag so that pairwise interactions could be inferred from the overlap of head capsules (electronic supplementary material, figure S10*b*,*c*). The proportion of time each individual spent in each box and the number of times each individual interacted with each other individual were then calculated using the python package *FortMyrmidon*. Individuals that were detected less than 2 × s.d. below the colony mean detection count were excluded from all analyses.

### (c) Statistical methods

All statistical analyses were conducted using *R* v. 4.3.2. The *t*-tests, Pearson’s correlations, one-sample proportion tests and ANOVAs were calculated in base *R* using functions *t.test*(), *cor.test*(), *aov*() and *prop.test*(), respectively. GLMM’s and generalized additive models were calculated using packages *lme4* and *mgcv,* respectively. Analysis code is available at: [75].

### (d) Soft community detection

Soft modularity, a measure of how well a proposed number of communities fits a given network, was calculated for *n*_communities_ = 2, 3, 4 and 5 for every network using a freely available implementation of the FacetNet algorithm (https://c4science.ch/source/facet_unil) [12,59,76]. For all species, soft modularity peaked when *n*_communities_ was set to 2, and so using this community number, the soft modularity was calculated for each of the 100 randomly rewired networks for all 25 colonies. The values observed for the original social contact networks were then compared to the distributions obtained from randomly rewired networks to test whether the observed networks were more modular than expected by chance (i.e. whether there was significant discontinuity in social interactions).

FacetNet additionally outputs community membership scores for each individual to each community, and we use membership to the foraging community (identified by the absence of the queen [20]) to define ‘social maturity’. This metric ranges from 0 to 1, with 0 indicating that an individual is maximally affiliated with the nurse community and 1 indicating that an individual is maximally affiliated with the forager community.

### (e) Summary metric calculation

— Node strength was calculated as the summed weight of all node edges using the function *strength* in R package ‘iGraph’ [77];
— node entropy was calculated using the function *Entropy* in R package ‘DescTools’ [78] over the vector of the number of interactions that a given node has with all other nodes, including 0 values where the two individuals did not interact;
— 90% home range sizes were calculated for every individual by ranking hexagons from the most visited to the least visited and then, starting with the most visited, sequentially adding hexagons to the home range until it covered at least 90% of total detection events for the individual; and
— the conservation in the identity of workers with which a queen interacts from one hour to the next was calculated as the number of workers that the queen interacted with in hour *t* that the queen had also interacted with in hour *t −* 1 divided by the total number of workers with which the queen interacted in hour *t −* 1.

## Data Availability

Data and code are available on Dryad [79]. Supplementary material is available online [80].
